# Prophylactic heminephrectomy for an asymptomatic, non-functioning moiety in pediatric duplex systems: is cancer prevention justified?

**DOI:** 10.3389/fped.2025.1722306

**Published:** 2025-12-18

**Authors:** Chenghao Zhanghuang, Na Long, Bing Yan

**Affiliations:** 1 Department of Urology, Kunming Children’s Hospital (Children’s Hospital Affiliated to Kunming Medical University), Kunming, China; 2 Yunnan Province Clinical Research Center for Children’s Health and Disease, Yunnan Key Laboratory of Children’s Major Disease Research, Kunming Pediatric Urology Minimally Invasive Surgery Technology Diagnosis and Treatment Center, Kunming, China; 3 Special Care Ward, Kunming Children’s Hospital (Children’s Hospital Affiliated to Kunming Medical University), Kunming, China

**Keywords:** duplex collecting system, heminephrectomy, prophylactic surgery, cancer prevention, wilms tumor, clinical surveillance

## Abstract

**Background:**

Duplication of the collecting system (DCS) is common. The management of asymptomatic, non-functioning moieties remains controversial because of a theoretical risk of malignancy, and prophylactic heminephrectomy is occasionally proposed despite the standard practice of reserving surgery for symptomatic complications.

**Objective:**

To critically appraise whether current evidence supports prophylactic heminephrectomy solely for cancer prevention in asymptomatic pediatric duplex systems.

**Methods:**

We conducted a focused narrative mini-review of PubMed (updated May 2025) and population-based cancer registries, concentrating on absolute malignancy risk and DCS-specific oncologic evidence.

**Evidence synthesis:**

Pediatric renal tumors are rare (Wilms tumor ≈7–10 per million children annually; pediatric renal cell carcinoma is even less frequent). The purported association between DCS and malignancy is based on six pediatric case reports (four Wilms tumors and two renal cell carcinomas). No denominator-based cohort data demonstrate an incidence above baseline. Mechanistic plausibility for carcinogenesis in a quiescent, uninfected, non-functioning moiety remains unproven. Although minimally invasive heminephrectomy is generally safe in experienced centers, complications such as bleeding, urinary leak, ureteral stump problems, and potential compromise of the preserved moiety are clinically relevant.

**Conclusions:**

Current evidence does not support routine prophylactic heminephrectomy for asymptomatic, non-functioning moieties in children. A conservative strategy with structured ultrasound surveillance at 6–12-month intervals and predefined surgical triggers is prudent and consistent with the available evidence.

## 1 Introduction

Duplication of the collecting system (DCS) is one of the most frequent congenital anomalies of the upper urinary tract. Children typically come to clinical attention because of associated abnormalities—ureterocele, ectopic ureter with continuous incontinence, vesicoureteral reflux (VUR), or severe hydroureteronephrosis—rather than because of the duplication itself (1, 2).

An “asymptomatic, non-functioning moiety” refers to a duplicated renal segment that demonstrates negligible cortical uptake on DMSA or non-visualization on MAG3 renography, in the absence of clinical symptoms such as infection, pain, or incontinence. Morphologically, such moieties may appear small, atrophic, or mildly dilated on ultrasound, but without evidence of ongoing obstruction, ureteral ectopia, or cortical thickening suggestive of active inflammation. When the upper pole is affected, mild calyceal or ureteral dilatation may represent a sequela of prenatal obstruction rather than a persisting obstructive process.

In contemporary pediatric urology practice, heminephrectomy is generally reserved for non-functioning or poorly functioning moieties when conventional indications are present: recurrent febrile urinary tract infections (UTIs) or pyonephrosis, progressive obstruction or megaureter with functional deterioration, ectopic ureter with incontinence, refractory calculi, or a painful or enlarging mass (3). By contrast, the suggestion that an asymptomatic, non-functioning moiety should be excised purely to reduce a hypothetical cancer risk is not supported by comparative data.

This mini-review addresses the specific question of whether an asymptomatic, non-functioning moiety in a pediatric duplex system should be removed solely to prevent malignancy. We summarize the baseline absolute risk of pediatric renal tumors, critically evaluate the limited case-level signal reported in duplex kidneys, and weigh potential oncologic benefit against surgical morbidity. The goal is to provide a pragmatic, evidence-aligned framework for clinical decision-making and surveillance rather than to propose a formal systematic guideline.

Proponents of prophylactic excision argue that eliminating a dysplastic or chronically obstructed segment may theoretically reduce future oncologic uncertainty; however, supporting pediatric data are lacking.

## 2 Epidemiology and absolute risk context

Anchoring decisions in absolute risk is essential. Across large population-based datasets and registry-linked analyses, Wilms tumor accounts for most pediatric renal cancers, with annual incidence commonly reported at approximately 7–10 per million children younger than 15 years (∼500–650 cases/year in the United States) (4–6). Pediatric RCC is far less common and typically affects adolescents (6). Upper-tract urothelial carcinoma in childhood is exceedingly rare and has mainly been described in small series or isolated case reports (7, 8).

These pediatric oncology data, derived from representative registries and reviews rather than from duplex-specific studies, provide the absolute-risk background against which the very limited case-level signal in duplex systems must be interpreted (Table 1).

**Table 1:** Representative population-based incidence of pediatric renal and upper-tract urothelial malignancies: absolute-risk context for duplex systems.

Tumor type	Approximate incidence in children	Key points relevant to duplex systems
Wilms tumor (nephroblastoma)	≈7–10 per million children per year; ∼90% of pediatric renal malignancies; peak 3–4 years (4, 5).	Common; usually normal kidneys. Congenital anomalies (incl. duplex) reported, but no duplex-specific incidence data.
Pediatric renal cell carcinoma (RCC)	≈0.1–0.3 per million per year; 3%–5% of pediatric renal tumors; mainly adolescents (6).	Biologically distinct from adult RCC. Only two pediatric RCC-in-duplex cases reported (14, 15); no evidence duplex increases RCC risk.
Upper-tract urothelial carcinoma (renal pelvis/ureter)	Extremely rare; most pediatric urothelial tumors are bladder primaries; upper tract mostly single case reports (7, 8).	Very few reports with duplex anatomy; true risk in duplex systems unknown but appears extremely low.

## 3 Biological plausibility—and pediatric limits

Beyond epidemiologic rarity, mechanistic plausibility for carcinogenesis in duplex systems remains weak. Arguments extrapolated from adult populations typically invoke a pathway of chronic irritation, obstruction and recurrent infection leading to metaplasia, dysplasia and carcinoma of the upper tract. In children, however, upper-tract urothelial cancers are intrinsically rare, and a quiescent, non-functioning moiety without ongoing infection or pressure may lack sustained carcinogenic stimuli (7, 8).

Importantly, none of the published pediatric DCS-associated malignancies provides longitudinal evidence that chronic inflammatory signaling within the duplicated moiety preceded oncogenesis. In addition, nearly all reported pediatric DCS-associated tumors presented with clinical symptoms or obvious radiologic lesions rather than arising in incidentally discovered, quiescent non-functioning moieties.

Taken together, biological plausibility alone is insufficient to justify prophylactic resection in an asymptomatic child.

## 4 Risk–benefit of prophylactic surgery in asymptomatic children

Minimally invasive heminephrectomy (laparoscopic or robotic) is well established and generally safe in experienced hands, but it is not risk-free. Reported complications include bleeding, urinary leakage, ureteral stump issues and potential compromise of the preserved moiety's blood supply or drainage (1–3, 9–13).

Given the very low baseline incidence of malignancy and the absence of cohort-level evidence that DCS confers an increased cancer risk, the expected oncologic benefit of prophylactic excision is extremely small, whereas perioperative morbidity—although infrequent—is concrete and immediate. On balance, a routine prophylactic strategy does not appear to offer a favorable risk–benefit profile in asymptomatic children.

When interpreting these data, it is also important to distinguish between registry age bands and clinical inclusion criteria. In this review, incidence figures in Table 1 are reported for children younger than 15 years, as this age band is used in most international cancer registries (e.g., IICC, SEER). In contrast, our inclusion criteria for individual reports followed common pediatric clinical practice and therefore encompassed patients younger than 18 years. These considerations are contextualized by the population-based incidence summarized in Table 1.

## 5 Literature search strategy

We performed a focused literature search in PubMed (MEDLINE) on 15 May 2025 to identify pediatric malignant tumors arising in duplicated renal systems. The strategy was designed to be narrow and anatomically specific rather than exhaustive for all pediatric renal tumors. No restriction on publication year was applied. The search was limited to human, English-language reports.

The following Boolean query was used in PubMed:

(“duplex kidney” [tiab] OR “duplex collecting system” [tiab] OR “duplex system” [tiab] OR “duplex ureter” [tiab] OR “duplicated collecting system” [tiab] OR “duplicated kidney” [tiab] OR “duplicated ureter” [tiab] OR “renal duplication” [tiab] OR “ureteral duplication” [tiab]) AND (“Wilms tumor” [tiab] OR “Wilms’ tumor” [tiab] OR nephroblastoma[tiab] OR “renal cell carcinoma” [tiab] OR “renal carcinoma” [tiab] OR “renal tumor” [tiab] OR “renal tumour” [tiab])

The search retrieved 23 records. After removal of duplicates (none in this set), all 23 titles and abstracts were screened. We included studies that met all of the following criteria: (i) at least one patient younger than 18 years; (ii) radiologic, intra-operative or pathologic confirmation of a duplex kidney or duplicated collecting system; and (iii) histologically confirmed renal parenchymal tumor or upper-tract urothelial carcinoma arising in the duplicated kidney.

We excluded adult-only reports, benign inflammatory lesions, non-duplicated kidneys, purely technical or imaging reports without duplex anatomy, and conference abstracts without full text. At the title/abstract level, 18 records were excluded because they involved adult patients, non-malignant inflammatory conditions (e.g., xanthogranulomatous pyelonephritis) or renal masses without duplicated anatomy. Five full-text case reports fulfilled all inclusion criteria and were retained in the final qualitative synthesis.

**Figure 1:**
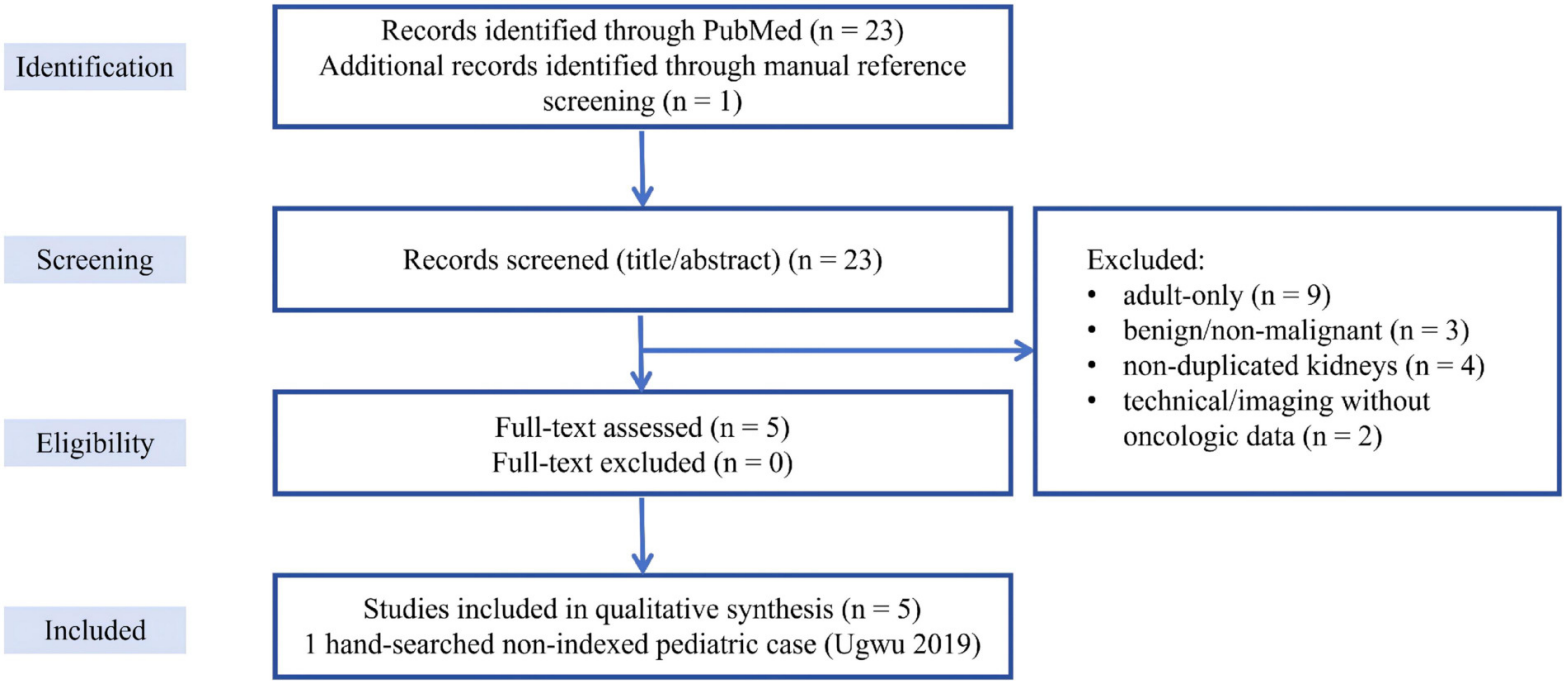
PRISMA-lite flow diagram for pediatric malignancies reported in duplicated collecting systems. PubMed search identified 23 records using an anatomically focused strategy (“duplex/duplicated kidney or collecting system” AND “Wilms tumor/nephroblastoma/renal cell carcinoma/renal tumor”). After title–abstract screening, 18 records were excluded (adult-only series, benign inflammatory lesions, non-duplicated kidneys, or purely technical reports), leaving 5 pediatric case reports that fulfilled all predefined criteria. One additional pediatric case published in a non-indexed regional journal was identified by hand-searching and is discussed narratively but not included in the PubMed flow.

In addition, one pediatric case of RCC in a duplex system published in a non-indexed regional journal was identified through hand-searching of reference lists and is discussed narratively but is not counted in the PubMed flow. The selection process is summarized in a PRISMA-lite flow diagram (Figure 1).

Because the available evidence consisted almost exclusively of isolated case reports, we did not attempt a formal meta-analysis. Broader background data on the incidence of pediatric renal tumors and on surgical outcomes in duplex systems were obtained from representative population-based and surgical series, which are cited narratively but are not included in the PRISMA diagram.

## 6 What the literature shows

No anatomopathological series or biopsy-confirmed analyses currently characterize the histological status of asymptomatic, non-functioning moieties in duplex systems. All published malignancy associations are based on postoperative or incidental pathological findings in symptomatic cases. Consequently, the existing evidence cannot determine whether quiescent, non-functioning moieties harbor premalignant changes.

The published association between DCS and malignancy is therefore almost entirely case-level. Five pediatric case reports were identified through our PubMed search, and one additional pediatric case (RCC in a 13-year-old girl with an ipsilateral duplex system) was retrieved from a non-indexed regional journal via hand-searching. Together, these six verified pediatric reports describe two RCC presentations—including an Xp11 translocation RCC in a 5-year-old—and four Wilms tumor presentations: one with preoperative rupture, one with extension into the duplex upper-pole ureter, one with a left duplex collecting system, and one bilateral Wilms tumor in the setting of a horseshoe kidney plus a left duplex kidney (14–19).

These cases demonstrate co-occurrence of malignancy and anatomical variants but do not quantify risk elevation compared with baseline. A PRISMA-lite schematic of our PubMed-based selection is provided in Figure 1, and the individual cases (including the hand-searched report) are summarized in Table 2.

**Table 2:** Published pediatric case reports of renal malignancies occurring in duplex collecting systems (case-level evidence only).

Reference	Age/sex	Tumor histology	Duplex anatomy	Management of renal lesion	Outcome/key notes
Alqarni et al. (14)	5-year-old girl	Renal cell carcinoma	Right duplex kidney; tumor in one moiety	Radical nephrectomy with lymph node sampling	Disease-free at short follow-up; duplex anatomy mainly increased technical difficulty.
Ugwu et al. (15)	13-year-old girl	Renal cell carcinoma	Ipsilateral duplex system; tumor in one moiety	Open nephroureterectomy of involved kidney	Good recovery; early disease-free follow-up. Coexistence considered likely coincidental.
Zhao et al. (16)	5-year-old girl	Wilms tumor with preoperative rupture	Left duplex kidney; inferior moiety tumor	Preoperative chemotherapy then radical nephrectomy; adjuvant chemotherapy	No recurrence at 11 months; illustrates staging and surgical challenges after rupture in duplex moiety.
Karnak et al. (17)	10-year-old girl	Wilms tumor with ureteral extension	Duplex system; extension into upper-pole ureter	Nephroureterectomy of involved moiety plus chemotherapy	Good outcome; highlights need to assess ureteral extension in duplex kidneys.
Kajbafzadeh et al. (18)	4-year-old boy	Wilms tumor	Left duplex collecting system; one component involved	Radical nephrectomy	Disease-free at ∼18 months; authors suggest association with duplex is probably coincidental.
Wu et al. (19)	Child (school-aged)	Bilateral Wilms tumor	Horseshoe kidney with left duplex system	Neoadjuvant chemotherapy; bilateral tumor resections; further surgery for recurrence	Recurrence at 6 months requiring re-resection; shows how complex anomalies can coexist with bilateral Wilms tumor.

Notably, all evidence linking DCS to malignancy derives from isolated reports and small descriptive series. This limitation reflects the rarity of both pediatric upper-tract cancers and duplex-system–associated malignancies, rather than selective citation. No denominator-based or population-level cohorts currently stratify malignancy incidence by renal anatomy. As a result, case reports remain the only available data source to illustrate possible co-occurrence, and their role is primarily hypothesis-generating rather than risk-estimating.

## 7 Pragmatic framework for decision-making and surveillance

Based on these findings, a pragmatic clinical framework is warranted. We recommend a structured, symptom-triggered strategy: (i) observe asymptomatic children with a non-functioning moiety and no ongoing infection/obstruction; (ii) institute renal/bladder ultrasonography at 6–12-month intervals to track moiety size, cortical thickness, and dilatation; (iii) escalate to DMSA or cross-sectional imaging only when clinical or sonographic change occurs; and (iv) adopt clear switch-to-surgery thresholds—first/recurrent febrile UTI/pyonephrosis, progressive obstruction/megaureter with deterioration, persistent incontinence from ectopic ureter, refractory calculi, or painful/enlarging mass (1–3, 9–13). Table 3 summarizes the evidence landscape underpinning this conservative, evidence-aligned stance.

**Table 3:** Evidence hierarchy supporting conservative management of asymptomatic, non-functioning moieties in pediatric duplex kidneys.

Evidence type	Representative examples	What this evidence shows	What this evidence cannot establish	Implications for managing asymptomatic duplex systems
Population-based incidence data for pediatric renal and urothelial tumors	PDQ Wilms guidance (4); ACCIS incidence data (5); pediatric RCC review (6); pediatric UC reports (7, 8)	Define overall incidence and age patterns of Wilms tumor, pediatric RCC and urothelial carcinoma in children.	Do not stratify tumor incidence by duplex anatomy.	Absolute malignancy risk in children is low; duplex anatomy alone does not justify prophylactic surgery.
Surgical series of duplex kidneys (non-oncologic indications)	Heminephrectomy/ureterocele series in duplex systems (1–3, 9–11, 13)	Describe indications and outcomes of surgery in duplex kidneys; no tumors observed during follow-up.	Sample sizes and follow-up are insufficient to exclude extremely rare tumors.	Support operating for clear urological indications, not for theoretical cancer prevention in asymptomatic duplex systems.
Pediatric case reports of malignancy in duplex systems	Six pediatric duplex + malignancy cases (14–19)	Show that Wilms tumor and RCC can arise in duplex kidneys and that anatomy can complicate imaging and surgery.	Cannot estimate absolute or relative risk and are prone to publication bias.	Support careful imaging and multidisciplinary planning when a mass is present, but not routine prophylactic heminephrectomy.
Clinical practice guidelines and narrative reviews	Wilms and pediatric kidney tumor PDQ (4); pediatric RCC overview (6); imaging review of anomalies (12)	Summarize consensus treatment of pediatric renal tumors and general principles for managing renal anomalies.	Do not provide duplex-specific surveillance algorithms or risk estimates.	Advocate managing asymptomatic duplex kidneys according to function and symptoms with standard imaging surveillance.

## 8 Conclusion

Current evidence does not support routine prophylactic heminephrectomy solely for cancer prevention in asymptomatic, non-functioning moieties of pediatric duplex systems. While an extremely rare incidental malignancy cannot be entirely excluded, the absence of denominator-based risk elevation and the presence of non-negligible surgical morbidity favor individualized observation with structured imaging follow-up and predefined triggers for intervention. Multi-institutional, anatomically annotated registries will be essential to quantify true malignancy risk and to inform future evidence-based guidance.
